# Assessing Educational Impact of Worldwide Webinar on Management of Myopia Progression in Children

**DOI:** 10.3390/ijerph21121661

**Published:** 2024-12-12

**Authors:** Meghal Gagrani, Jonathan Heston, Daisy Godts, David Granet, Dominique Bremond-Gignac, Ramesh Kekunnaya, Richard W. Hertle, Seo Wei Leo, Ken K. Nischal

**Affiliations:** 1 Division of Pediatric Ophthalmology and Strabismus, UPMC Children’s Hospital of Pittsburgh, Pittsburgh, PA 15224, USA; gagranim@upmc.edu; 2 The Fundingsland Group, Gilroy, CA 95020, USA; jon@tfgeducation.com; 3 Department of Ophthalmology, Antwerp University Hospital, 2650 Antwerp, Belgium; daisy.godts@uza.be; 4 Viterbi Family Department of Ophthalmology, Ratner Children’s Eye Center of the Shiley Eye Institute, University of California San Diego, La Jolla, CA 92093, USA; dgranet@gmail.com; 5 Ophthalmology Department, Necker-Enfants Malades University Hospital, AP-HP, Paris Cité University, 75015 Paris, France; dominique.bremond@aphp.fr; 6 Child Sight Institute, Infor Myopia Center & Center for Tech Innovation, L V Prasad Eye Institute, Hyderabad 500034, India; drrk123@gmail.com; 7 Department of Pediatric Ophthalmology, Akron Children’s Hospital, Akron, OH 44308, USA; rhertle@akronchildrens.org; 8 Dr Leo Adult & Paediatric Eye Specialist Ltd., Singapore 228510, Singapore; drleoseowei@gmail.com

**Keywords:** myopia, medical education, webinar, pediatric eye care

## Abstract

Objective: To assess the educational impact of a worldwide webinar approach to myopia progression management in children <8 years and 8–12 years old. Design: Cross-sectional study. Methods: A self-administered survey was conducted for attendees of a 3 h worldwide webinar held in two parts on consecutive days on the management of myopia progression in children. The survey was administered before, immediately after completion of the webinar, and 8 weeks later; responses were recorded on a Likert scale. Questions were posed to assess (a) the confidence of attendees in managing myopia in children <12 years old, (b) attendees’ understanding of latest treatment options, (c) any improvement in attendees’ knowledge after the webinar, and (d) any changes made to practice 8 weeks after the webinar. Pre- and post-responses were analyzed using an unpaired two-tailed *t*-test. Results: The webinar had 701 and 606 global attendees on the first and second days, respectively. Based on a comparison of contact information, 372 attendees participated on days 1 and 2, meaning 288 and 233 participants attended only day 1 and day 2, respectively. There was a significant increase in the percentage of attendees who were “very confident” in managing myopia after the webinar (*p* < 0.05). Ninety-nine attendees completed the survey at 8 weeks. Of these, 76% believed that the webinar had “very significantly” or “significantly” improved their ability to manage pediatric myopia and 91% had implemented or intended to implement a change in their practice. The respondents who did not implement a change identified cost and patient compliance as the common barriers. Conclusion: There is a tsunami of research and management options in the field of myopia management at present. We demonstrate that an effective way of disseminating information and education about myopia management is a pre-designed comprehensive webinar held over two consecutive days. There is evidence that such a webinar may also influence a change in clinical practice.

## 1. Background

Myopia is on the rise among children and adolescents, and it has earned the status of a growing epidemic in recent years. The prevalence of myopia has surged globally, with some regions reporting rates as high as 80–90% among teenagers and young adults [1]. It is estimated that approximately 50% of the world’s population will be myopic by 2050, with as much as 10% with high myopia [2]. This may directly cause an increase in low vision and blindness due to complications associated with high myopia that may result in pathologic ocular changes and potentially irreversible blindness [3]. Therefore, myopia has become a worldwide public health problem and there is a need to reduce the myopia progression and prevalence of high myopia [4]. Extensive research and trials are ongoing worldwide, exploring various treatment options for myopia progression management. Behavioral interventions for myopia control are well described [5] and have led to policy changes in the schools in certain parts of the world [6,7]. However, in the last few years, various other strategies have evolved to prevent myopia progression. These include topical pharmacological options such as low-dose atropine, specially designed spectacle lenses, specially designed contact lenses, and orthokeratology. Currently, myopia management in children lacks standardization, despite the existence of several published position papers. Moreover, the knowledge, accessibility, and the acceptance of various myopia control methods vary depending on the geographic location [8].

A global survey conducted in 2019 found that 52% of eye care practitioners still prescribed single-vision spectacles or contact lenses as the primary mode of correction for myopic patients, and orthokeratology was thought to be the most efficacious intervention method [9]. The main reasons for their reluctance to prescribe alternatives to single-vision refractive corrections were increased cost (20.6%) and inadequate information (17.6%) [9]. The same authors recently published an updated report where the survey was undertaken again in 2022, and this time combination therapy was perceived to be the most effective treatment strategy across the world; however, only 2–5% of the practitioners practiced combination therapy [8].

A more recent survey was conducted among eye care practitioners in Singapore, a country with one of the highest prevalence of both myopia and high myopia [10]. It was revealed that 58% of practitioners face the need for more education on interventions for myopia control in order to use them comfortably [11]. There is a need for increased awareness among care providers and change in practice patterns.

The World Society of Paediatric Ophthalmology and Strabismus (WSPOS) a registered charity (Charity Registration Number: 1144806) conducted a survey in the autumn of 2022 to assess the educational needs for myopia management and subsequently conducted a 3 h online webinar to discuss updates on myopia management in children in November 2022. The webinar was conducted over two consecutive days with each session lasting 90 min. The primary purpose of the current study was to assess the educational impact of this webinar on the attendees’ knowledge and clinical practice.

## 2. Methods

This cross-sectional study was conducted using all deidentified responses and did not involve any human subjects research and therefore was exempted by the Institutional Review Board of University of Pittsburgh School of Medicine. An investigative 11-question survey was conducted by WSPOS in November 2022 to gather data regarding the current practice patterns to treat myopia progression used by pediatric eye care providers, including pediatric ophthalmologists, general ophthalmologists, and optometrists across the world, and to assess the need for further education/awareness. This survey was sent online to all the members of WSPOS prior to the webinar and is referred to the investigative survey.

A 3 h myopia management webinar spread over two days was conducted online later in November 2022. The webinar content is shown in Table 1. It was free to attend for all who had registered for it and was additionally recorded and made available to view at a later date on YouTube (https://youtu.be/gAXxvJqu3h8?si=njbkmXNNIuTCLEAq, https://www.youtube.com/watch?v=gAXxvJqu3h8, accessed on 10 November 2024). A self-administered assessment survey (Table 2) was conducted in English at the beginning of the webinar to gauge the confidence of the audience in managing myopia in children <8 years old and 8–12 years old along with their understanding of the latest treatment options for myopia management. The responses were recorded on a 3–5-point Likert scale as shown in Table 2.

A similar assessment survey was presented again at the end of the webinar, and responses were recorded. Questions 1 and 2 of the assessment survey were posed on both days, question 3 was specific for day 1, and question 4 was specific for day 2, as shown in Table 2. At 8 weeks after the webinar, the same survey was posed again via an email sent to the registered email addresses, and respondents were also asked if they implemented a change in their practice of myopia management or if they intended to do so in the near future. If there was no change in the practice pattern, any barriers preventing them from changing their practice was also evaluated.

Statistical analysis: For questions on a Likert scale (i.e., confidence or understanding), each response was converted to a 1 to M scale, where M represents the number of response options. The most negative valence response is assigned value 1 and the most positive is assigned value M (i.e., not confident = 1; very confident = 4). From here, the means and standard deviations of response values were calculated for each question at each time point. An unpaired, 2-tailed *t*-test was then used to compare pre vs. post and pre vs. 8 weeks post, using the mean, standard deviation, and N of response values at each time point.

For questions that compared proportions and did not report on measures that are not on a Likert scale, statistical significance was tested following the methods used in Kohnen et al., 2022 [12]. In brief, Z scores were calculated based on the proportions and total N for each group in the comparison pre and post or pre and 8 weeks post. The resulting Z score was used to derive a *p* value using the following online calculator: https://www.socscistatistics.com/tests/ztest/default2.aspx (accessed 30 September 2023).

## 3. Results

The initial investigative survey to assess educational needs was completed by 326 practitioners from 64 countries. Seventy-six percent of the respondents saw more than 10 pediatric myopia patients in their practice in a month and on average 26 patients per month. Overall, 89% of respondents reported that the number of pediatric myopia patients in their practice has increased in the last 2 years, with 55% reporting a substantial increase. The strategies used by respondents for myopia progression management included behavioral modification, pharmaceutical options, spectacle lenses such as defocus incorporated multiple segment spectacle lenses, highly aspherical lenslet spectacle lenses, orthokeratology, and multifocal soft contact lenses.

**Figure 1:**
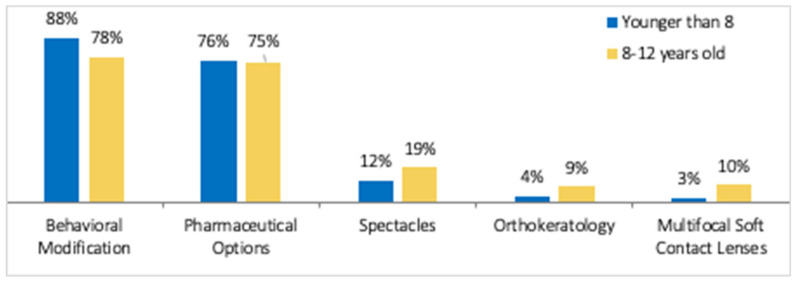
The most common strategies used by respondents for treating myopia progression in patients ≤ 12 years of age as found in the investigative survey.

The top two strategies used in both age groups (<8 years old and 8–12 years old) were behavioral modification and pharmaceutical options. The responses are shown in Figure 1.

The most common behavioral modification recommended was spending more time outdoors (57%) and reduced time spent on devices (36%).

**Figure 2:**
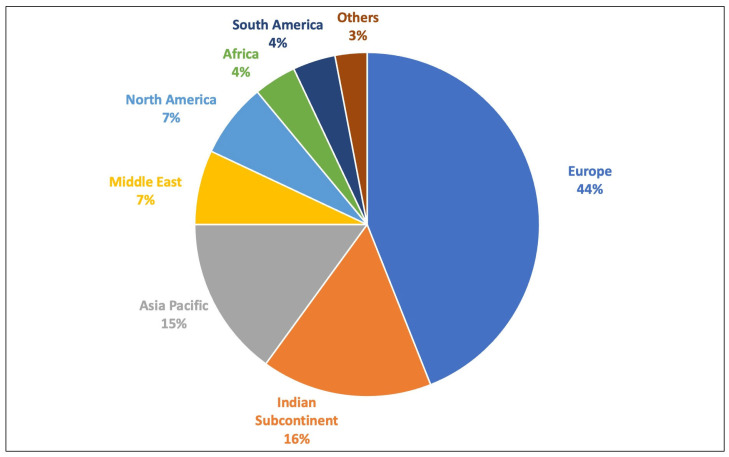
Pie chart representing the geographic distribution of the participants of the myopia management webinar.

The webinar had 701 global attendees on the first day and 606 global attendees on the second day. Of these, 660 and 605 attendees provided their contact info, with the remaining attendees participating anonymously. Based on a comparison of contact info, 372 attendees participated on days 1 and 2, meaning 288 and 233 participants attended only day 1 and day 2, respectively. The maximum participation was from Europe (43%), followed by the Indian subcontinent (16%), with representation from all over the world (Figure 2).

**Figure 3:**
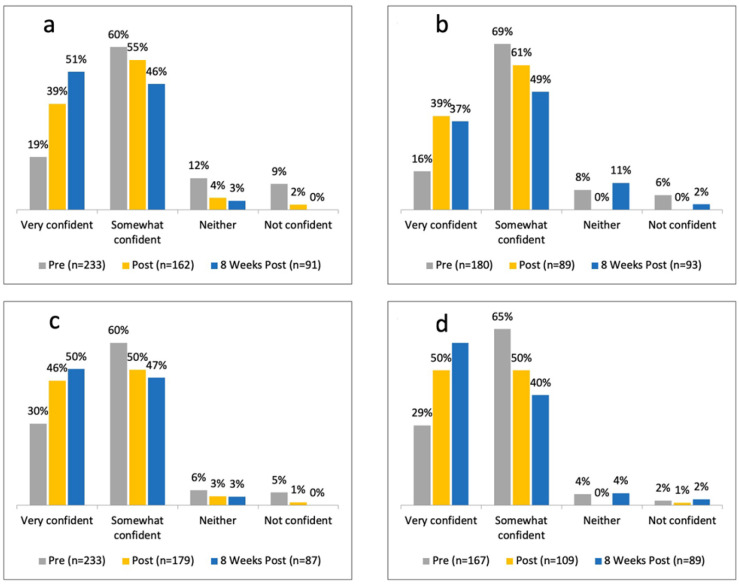
Graph showing the confidence level of respondents in myopia progression management before the webinar, immediately after the webinar, and 8 weeks later in patients (**a**) <8 years of age on first day (**b**) <8 years of age on second day (**c**) 8–12 years of age on first day (**d**) 8–12 years of age on second day.

At the beginning of the first webinar using the assessment survey (Table 2), 19% of the respondents were “very confident” in managing myopia progression in children <8 years of age, and 30% of respondents were “very confident” in managing patients 8–12 years of age. At the end of the first webinar, there was a significant increase in the respondents who were “very confident” in the myopia management, 51% in <8 years old (*p* = 6.51 × 10^−9^) and 46% in 8–12 years old (*p* = 0.0005), respectively (Figure 3a, 3c).

At the beginning of the second webinar using the assessment survey (Table 2), 16% of the respondents were “very confident” in managing myopia progression in children <8 years of age, and 29% of respondents were “very confident” in managing patients 8–12 years of age. At the end of the second webinar, there was a significant increase in the respondents who were “very confident” in the myopia management, 39% in <8 years old (*p* <0.05) and 50% in 8–12 years old (*p* = 0.0005), respectively (Figure 3b,d).

There was a further increase in this percentage at the 8-week follow-up assessment survey, as shown in Figure 3, which was statistically significant for both <8 year olds (*p* < 0.001) and 8–12 year olds (*p* < 0.05).

**Figure 4:**
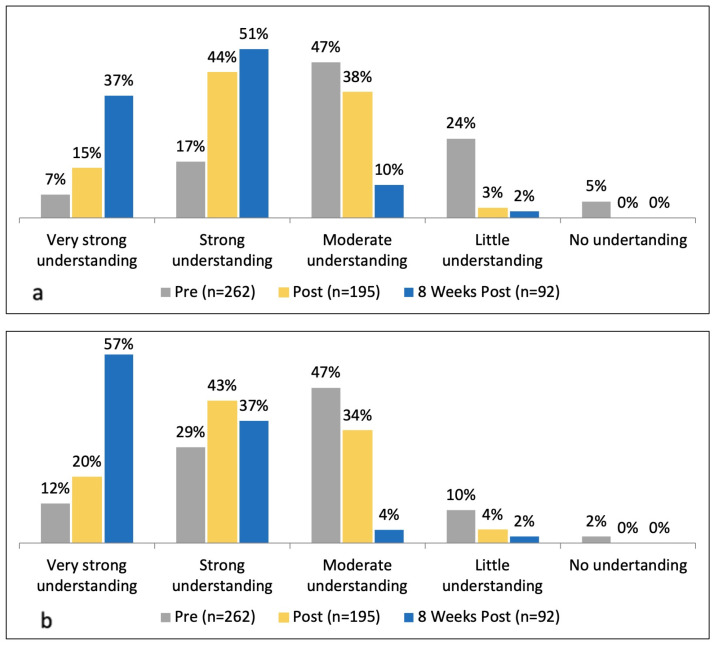
Graph showing the level of understanding of various myopia treatment options for management of pediatric myopia before the webinar, immediately after, and 8 weeks late. (**a**) Orthokeratology and multifocal soft contact lenses; (**b**) spectacles and pharmaceutical options.

At the beginning of the webinar, 24% of the respondents had a strong or very strong understanding of the latest treatment options for myopia management, including orthokeratology and multifocal soft contact lenses. This increased to 59% after the symposium and 88% at 8 weeks after the program (Figure 4a). A higher percentage of respondents (41%) had a strong or very strong understanding of spectacles and pharmaceutical options, and, similar to the other treatment modalities, saw an increase to 63% after the webinar (Figure 4b).

**Figure 5:**
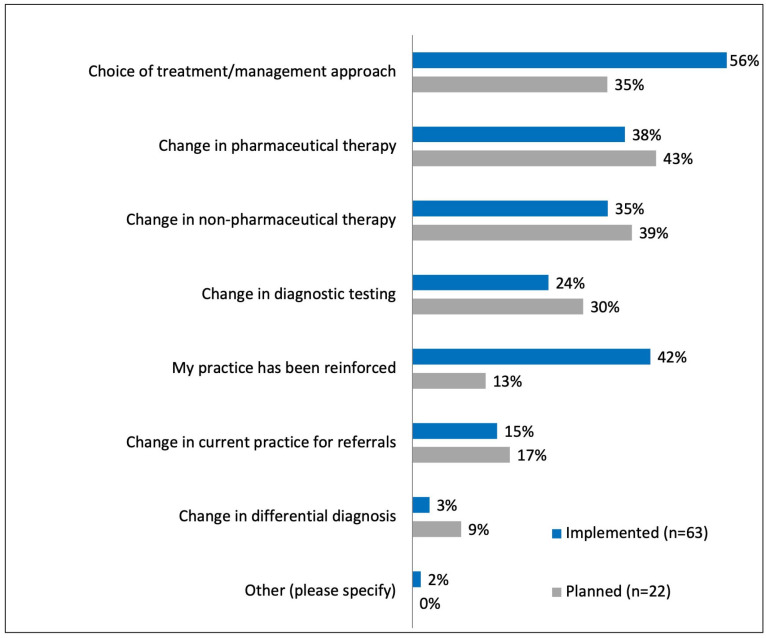
Graph showing the changes implemented or planned in practice 8 weeks after the webinar.

Ninety-nine attendees completed the survey eight weeks after the webinar. Seventy-six percent of the respondents at 8 weeks believed that what they learned in the webinar has “very significantly” or “significantly” improved their ability to manage pediatric myopia progression in their practice. Of these, 91% (90 out of 99) of respondents had either implemented a change to their practice (67%) or planned to do so (23%). The changes implemented or intended to be implemented are shown in Figure 5.

The remaining 9% of the respondents, who had not implemented a change or intended to do so, identified cost (22%) and patient compliance issues (22%) as the most common factors preventing them from making a change. Eleven percent of respondents identified a lack of experience as a barrier to change. Ninety-nine percent indicated that they would like to participate in future education programs on similar topics.

## 4. Discussion

Myopia is a growing epidemic, as discussed earlier, and this was acknowledged by the participants of our survey, with more than 50% of the respondents reporting a substantial increase in their practice in the last 2 years. Whether this is because of increased parental awareness or an actual increase in myopia prevalence cannot be judged from this study, but other epidemiological studies suggest that it is the latter [2,13,14]. This calls for increased awareness among caregivers and parents about the newly emerging and ever-evolving treatment options. However, translation of evidence-based medicine to evidence-based practice is challenging [15], and medical education has been identified as one of the major strategies to incorporate newly evolving evidence into clinical practice [15,16].

While there are many articles and symposia about managing myopia progression, as far as we are aware, this is the first study to measure the effect of such education on ophthalmic practitioners using re-presentation of the same assessment survey immediately before, immediately after, and 8 weeks after a two-day educational webinar lasting a total of 3 h.

The WSPOS investigative survey identified that behavioral modification and pharmacological treatment were the most common treatment options being used for myopia control in children, being used by more than 75% of the respondents, and less than 20% used the other available strategies. This proportion is similar to other worldwide surveys conducted previously [17,18,19]. Leshno et al. [17] conducted a survey in 2019 and found almost all respondents (*n* = 794) used some form of intervention for myopia control, with behavioral modification being the most common form of intervention across the globe. These surveys were cross-sectional and did not measure the effect of an educational intervention.

The COVID-19 pandemic has accelerated the rise of internet-based education, which has transformed learning and is definitely here to stay [20]. WSPOS started holding educational webinars during the lockdown of the pandemic, with the first on 25 April 2020, and had held 51 webinars until August 2022, utilizing an established platform. We used this platform to conduct a worldwide webinar to increase awareness about all the new available strategies for myopia management. Additionally, this report represents one of the rare opportunities to evaluate the impact of internet-based global education. Post-pandemic, this is the first worldwide webinar, to the best of our knowledge, where the impact on global practice patterns was analyzed.

The webinar was attended by pediatric eye care providers (ophthalmologists, orthoptists, and optometrists) from all six continents, with the most participation from Europe (44%). The disparity in global participation could be related to the availability of options for myopia control in different parts of the world, with most of these options being available in the European countries.

Prior to the webinar, the assessment survey revealed a relatively small number of participants who were “very confident” in managing myopia in children 8–12 years of age (30%) as compared to <8 years old (18%). However, there was an extremely significant increase in the number of respondents who were “very confident” in treating myopia progression in <8 years and 8–12 years old, 8 weeks after the program: 32% point and 25% point increase, respectively.

Although our initial response rate to the survey (day 1, Figure 3a) was relatively high compared to similar studies in other areas of medicine [21,22,23], the re-survey response rate at 8 weeks was low (14%). The reduced survey response rate can be attributed to the differences in survey presentation methods. Although there are no specific cut-offs for adequate response rates, online surveys presented during webinars typically have higher response rates compared to email surveys [24,25]. However, we have been unable to find any other study where a re-survey had been conducted for evaluation of medical intervention. While pre- and immediate post-webinar survey responses have been reported in other areas of medicine, as far as we are aware, this is the first study to report, pre-, immediately after, and 8-week survey responses.

About 75% of the participants who responded to the survey at 8 weeks confirmed that participation in the webinar(s) improved their ability to treat myopia progression in their practice. This demonstrates the educational impact of the global webinar to create a change in practice patterns, as shown in Figure 5. Sixty-one percent of the respondents had already implemented a change in their practice, most commonly by changing the choice of treatment or management approach.

It has been well recognized that individualized management of myopia [4], with tailoring the interventions to specific patient profiles, is the preferred approach to myopia treatment given the environmental [1,5,26] and genetic factors at play [27,28]. In the recent worldwide survey conducted by Wolffsohn et al. [8], combination therapy was perceived as the most effective treatment strategy, yet, only a small proportion of respondents indicated practicing that approach. Education regarding available treatment options can help provide a tailored approach to the specific population a clinician is responsible for (depending on the geographical, ethnicity, school education, etc.) and consequently improve patient outcomes.

Respondents who did not make a change to their practice specifically identified cost and poor patient compliance as the limiting factors. Cost has previously also been identified as a limiting factor in several parts of the world, especially developing countries [28]. This stresses the importance and awareness of access to and equity in health care. Education of caregivers and patients is a crucial first step in improving access to newer treatment modalities globally, as has been demonstrated [8,9,29], and healthcare providers play a key role in guiding them through available treatment options.

Moore’s seven-level outcomes model [30] describes in detail the importance of integrating planning of educational activities with assessment of its impact. Moore’s recommended approach was specifically used in framing the questions evaluating the educational impact and usefulness of the webinar(s). For example, Moore et al. [30]. describe that planning an educational activity begins with identifying the gap in knowledge; therefore, the pre-webinar survey was conducted. Although traditional articles are a well-established method for disseminating medical information, they may have limited reach compared to webinars and social media, as they are often limited to the readership of the specific journal in which they are published [31]. Webinars have the advantage of reaching a larger audience and the convenience from attending anywhere [32]. Webinars have been shown to be effective in information sharing, idea exchange, and connection development, especially since the COVID-19 pandemic. To the best of our knowledge, this is the first study reporting the educational value of webinars and change in practice patterns for myopia management.

We acknowledge the limitations of our study. We analyze self-reported outcomes given it would be very difficult to objectively assess change in practice patterns on a worldwide scale, and thus accept the inherent limitations that it imposes. There may be a <50% response bias of the pre-webinar respondents who completed the 8-week survey, including the possibility that people who gained more from the webinar responded to the 8-week survey. All survey questions were not answered by all attendees, and therefore, the number of respondents for different questions may have differed adding to the bias. Larger pragmatic studies are needed to look at the translational value of these educational activities.

In conclusion, this report shows that there is a significant gap in knowledge in treating myopia progression in children, and webinar(s) based upon sound educational principles can and did improve the confidence of the attendees in managing this condition, resulting in a change in practice patterns. Importantly, this demonstrated that a successful approach is needed to impact areas where health access and equity must be improved.

## Figures and Tables

**Table 1:** Program of the two-day webinar for myopia management and the timeline for audience questions.

**Day 1 Program: 19 November 2022**
Part 1: Defining the problem
1.1 *Audience response questions*
1.2 The global epidemic of progressive myopia
1.3 The real problem: more high myopes
1.4 Changing views on the impact of Myopia

Part 2a: Treatment strategies to slow myopia progression
2.1 First-line treatment: behavioral modification
2.2 Overnight orthokeratology
2.3 New daily wear contact lenses designed for myopia control
2.4 FDA study perspective on contact lenses for myopia control
2.5 *Audience response questions*
**Day 2 program 20 November 2022**
Part 2b: Treatment strategies to slow myopia progression
2.6 *Audience response questions*
2.7 Preventing axial length elongation with a pair of spectacles: Can it be done?
2.8 The science behind pharmacological modulation for myopia control

Part 3: Strategies for integrating myopia management into the practice
3.1 Reviewing the latest WSPOS guidelines
3.2 Managing your professional network for earlier intervention
3.3 Best practices for patient education
3.4 *Audience response questions*

**Table 2:** Questions presented in the assessment survey conducted before the seminar, immediately after, and 8 weeks later.

S. No	Question	Options
1	How confident are you in your ability to manage and treat myopia progression in a patient <8 years of age? (Day 1 and 2)	a. Very confident b. Somewhat confident c. Neither d. Not confident
2	How confident are you in your ability to manage and treat myopia progression in a patient between 8–12 years of age? (Day 1 and 2)	a. Very confident b. Somewhat confident c. Neither d. Not confident
3	How strong is your understanding of the latest treatment options for myopia management including orthokeratology and multifocal soft contact lenses? (Day 1 only)	a. Very strong understanding b. Strong understanding c. Moderate understanding d. Little understanding e. No understanding
4	How strong is your understanding of the latest treatment options for myopia management including spectacles and pharmaceutical options? (Day 2 only)	a. Very strong understanding b. Strong understanding c. Moderate understanding d. Little understanding, or e. No understanding
5 *	How significantly do you believe what you have learned today has improved your ability to manage myopia progression in paediatric patients in your practice? * (asked only after the webinar on Day 1 and 2 and at 8 weeks)	a. Very significantly b. Significantly c. Somewhat significantly d. No change

## Data Availability

Data can be made available on request.
